# Accuracy of smartwatches in predicting distance running performance

**DOI:** 10.3389/fspor.2025.1517632

**Published:** 2025-01-29

**Authors:** Jiansong Dai, Gangrui Chen, Zhonghe Gu, Yuxuan Qi, Kai Xu

**Affiliations:** ^1^ Department of Sports and Health, Nanjing Sport Institute, Nanjing, China; ^2^ Sport Science Research Institute, Nanjing Sport Institute, Nanjing, China; ^3^ School of Physical Education, Performance and Sport Leadership, Springfield College, Springfield, MA, United States

**Keywords:** smartwatch, running, performance prediction, amateur runners, accuracy

## Abstract

**Objective:**

This study examined the accuracy of smartwatches in predicting running performance.

**Methods:**

A total of 154 amateur runners (123 males and 31 females) were recruited. After wearing the HUAWEI WATCH GT Runner for a minimum of six weeks, the runners' actual completion times for 5 km, 10 km, and half marathon distances were measured, resulting in 288 test instances. The predicted completion times for the same distances displayed on the watch on the test day were recorded simultaneously.

**Results:**

The actual and predicted performances for the 5, 10, and 21.1 km distances were highly correlated, with *r* ≥ 0.95 (*p* < 0.001) and *r*^2^ ≥ 0.9 for all three distances, an error rate between the measured and predicted values of less than 3%, and intraclass correlation coefficient ≥0.9. The bias ± 95% limits of agreement were −20.4 ± 44.2 s for 5 km, 4.1 ± 299.1 s for 10 km, and 143.8 ± 400.4 s for the half marathon.

**Conclusions:**

This study confirmed that the smartwatch exhibits high precision in predicting 5 km, 10 km, and half marathon performances, with an accuracy exceeding 97%. The performance prediction features of smartwatches can effectively guide amateur runners in setting reasonable competition goals and preparing for races.

## 1 Introduction

Running has several health benefits, including reduced all-cause and cardiovascular disease mortality (1). This has led to the global popularity of running and marathons. The number of marathons held in China increased from 12 in 2010 to 53 in 2014 and rapidly grew to approximately 1,100 by 2017, with nearly 5 million participants. The number of participants increased by over 2.2 million between 2016 and 2017. By 2019, the number of events had risen to 1,900, attracting approximately 7 million participants (2). This has sparked tremendous interest in running performance prediction among many individuals, from competitive athletes to amateur enthusiasts, because it can help them prepare for competitions and train more effectively.

Numerous studies have demonstrated that running performance can be predicted through single or combined measures of anthropometric and physiological markers (3–6). In early laboratory conditions, running performance was highly correlated with treadmill speed at peak maximal oxygen uptake (*r* = –0.88 to −0.94); lactate threshold running speed (*r* = –0.80 to −0.92), and VO2max% at 16 km/h (*r* = 0.76–0.90); and VO2max (*r* = 0.55–0.86). Additionally, the time taken to complete the 10 km or 21.1 km races was a predictive marker for marathon and ultramarathon performances (*r* = 0.91–0.97), suggesting that medium-long distance running performances are also highly correlated with marathon or longer distance race performance (7). One study used a linear graph model based on the time taken by runners to complete two different distances, facilitating the prediction of performance at various distances from the 10 km race to marathons. The results revealed correlation coefficients of 0.89 and 0.97 for the 10 and 20 km times, respectively, with marathons (8, 9). Other studies have found that the total duration of progressively increasing load testing on a treadmill correlates with marathon performance (*r^2^* = 0.45) (5). However, an outdoor 12-minute run test (Cooper test) was found to predict half-marathon performance more accurately than progressive increases in laboratory-based maximal oxygen uptake tests (outdoor test: *r^2^* = 0.873, indoor test: *r^2^* = 0.769) (10). Given that running involves long-duration endurance activity against one's body weight, a lighter weight and lower fat content are evidently beneficial for improving performance. Hence, some studies have established sex-specific linear prediction models based on body fat percentage and everyday training running speed (males: *r^2^* = 0.42, females: *r^2^* = 0.68) (11). Other studies incorporated parameters such as body fat percentage, running speed, and weekly training volume into a linear model, achieving an *r^2^* of 0.81 (12).

Besides the anthropometric and physiological indicators, training parameters such as the frequency of training, total running volume, average volume per run, maximum weekly training distance, and training intensity also influence marathon performance. Research suggests that the maximum weekly training distance is an important predictor of marathon performance among amateur runners (13). In long-distance races between 10 and 90 km, runners who ran more than 100 km per week completed the race significantly faster than those with lower weekly running volumes (14). Other studies have found that if the maximum training volume in daily training exceeds 21 km and the average training volume exceeds 10 km, the monthly training volume becomes the most important factor in predicting marathon performance. However, if these conditions are not met, the monthly training volume is not related to marathon performance, suggesting that the monthly training volume only significantly affects marathon performance when daily training distances reach a certain level (15). Overall, runners who train more frequently with higher weekly training volumes and longer training durations tend to perform better in races (16). Elite marathon runners have significantly higher total weekly running volumes and training speeds than average marathon runners (14) making these the core reasons for their superior performance.

Although marathon performance can be predicted using anthropometric and physiological indicators (7, 12), these measures require strict testing conditions and expensive equipment, limiting their application to a small segment of the population. Consequently, applying these prediction models to amateur runners is difficult. However, training parameters are easier for amateur runners to obtain. Extensive research has confirmed that training parameters are crucial for predicting marathon performance. Furthermore, running tests conducted in natural environments may provide more accurate predictions of running performance than laboratory tests (10).

With the rapid development of smart wearable devices, wristbands and smartwatches are widely used in the fitness field and have been validated for evaluating physiological indicators such as maximal oxygen uptake (VO2max), maximum heart rate (HRmax), energy expenditure (EE), and heart rate variability (HRV). Smartwatches are prevalent among amateur runners. They can accurately record training parameters, such as distance, time, and running speed, and physiological parameters, such as heart rate, while allowing for long-term data accumulation. Many running watch brands have developed running performance prediction functions based on training and physiological parameters. However, no publicly published studies have validated the accuracy of running performance predictions using these smartwatches. In this study, we selected the HUAWEI WATCH GT Runner as the device to be validated. By comparing the predicted running performance of amateur runners using the HUAWEI WATCH GT Runner with their actual running performance, we assessed the accuracy and error level of the watch's performance prediction function. We hypothesized that smartwatch-predicted running performance would be highly consistent with the actual running performance.

## 2 Materials and methods

### 2.1 Participants

Between January 2021 and November 2022, 154 amateur runners (123 males and 31 females) participated in this study. All participants had experience running and participating in marathons. Participants' basic information is presented in Table 1. This study was approved by the Ethics Committee of Nanjing Sports Institute in accordance with the Declaration of Helsinki. The participants were informed about the experimental plan, procedure, and related testing requirements, and were asked to sign a written informed consent form before testing. To improve the quality and accuracy of the test data, the participants were asked not to engage in high-intensity exercise or consume food containing caffeine or alcohol 48 h before testing.

**Table 1:** Basic information of participants.

	Male (*N* = 123)	Female (*N* = 31)
Age (years)	34.2 ± 4.6	30.5 ± 2.3
Height (cm)	172.6 ± 5.2	159.3 ± 3.5
Weight (kg)	67.9 ± 8.0	54.3 ± 4.5
BMI [body mass (kg)·height (m)^−2^]	22.8 ± 2.4	21.4 ± 2.0
Years of running (years)	4.6 ± 2.4	3.7 ± 1.8
Average monthly running distance (km)	155.0 ± 70.1	82.6 ± 25.4
Average training pace (sec/km)	339.5 ± 46.4	390.9 ± 34.5
Average monthly running time (h)	15.9 ± 8.6	10.0 ± 4.4
Average half-marathon best time (min)	102 ± 19	131 ± 20
Average marathon best time (min)	196 ± 55	266 ± 28
5 KM Measured Performance maximum heart rate (%)	98.69 ± 0.98	98.85 ± 1.02
10 KM Measured Performance maximum heart rate (%)	94.85 ± 1.29	94.68 ± 1.38
21.1 KM Measured Performance maximum heart rate (%)	84.89 ± 2.84	84.41 ± 2.68
5 KM Measured Performance RPE	17.89 ± 0.68	17.90 ± 0.10
10 KM Measured Performance RPE	17.97 ± 0.58	17.96 ± 0.59
21.1 KM Measured Performance RPE	18.09 ± 0.53	17.85 ± 0.65

Values are mean ± SD.

### 2.2 Experimental procedure

The study protocol was approved by the Institutional Review Board of the Bioethical Committee of the Nanjing Sports Institute (RT-2021-03) and met the requirements of the Declaration of Helsinki. HUAWEI WATCH GT Runner devices were distributed to 154 participants who wore them for a period ranging from three months to two years. During this period, the runners maintained their normal training habits without any intervention in their routine. For each running session, the watch was set to record the relevant parameters such as heart rate, time, and pace. At the end of each session, the data were saved, uploaded, and updated using a Bluetooth-connected application. The predicted performance was recorded on the day of the test and before the start of the measured performance test. The predicted performance refers to the smartwatch's prediction of the time it takes the participant to complete 5, 10, and 21.1 km. The measured performance refers to the time taken by the participant to complete 5, 10, and 21.1 km with maximum effort.

To obtain more reliable predictive scores from the participants, they were required to wear the device for a minimum of six weeks. We organized periodic tests for 5 km, 10 km, and half-marathon distances. A total of 154 participants underwent 288 testing sessions throughout the study period, with variations in the number of completed tests and distances covered by each participant. The actual number of test sessions per participant ranged from one to six. Prior to the running test, it was ensured that the participants were not physically fatigued, had rested for at least two consecutive days prior to the test, had not consumed caffeinated or alcoholic food, and had completed only one distance at a time, with a three-month interval between two adjacent tests. Prior to each test, the predicted scores displayed on the participants' watches were recorded. The half-marathon test was conducted outdoors on a known half-marathon route, and the 5 and 10 km tests were conducted on a standard 400 m track. The participants were required to exert their full effort during the tests, and their actual performance was recorded. Immediately following the test, the participants were asked to rate their perceived exertion (RPE_6–20_) (17). In the RPE6–20 scale, a 6 is labeled as “very easy” and a 20 is labeled as “total exhaustion.” All participants wore the HUAWEI WATCH GT Runner smartwatch during the run to track their exercise heart rate. The maximum heart rate for each participant was calculated by subtracting their age from 220. The intensity level was calculated by dividing the average heart rate during the event by the maximum heart rate. The actual tests were conducted in the morning, when the temperature was 16–24 degrees Celsius and the relative humidity did not exceed 50%.

### 2.3 Smartwatch

In this study, the smartwatch model of HUAWEI WATCH GT Runner was selected. This smartwatch is based on the HUAWEI TruSportTM scientific training system, which encompasses a comprehensive set of running performance algorithms. By leveraging smart wearable devices such as watches, accessories, and mobile management software (APP), as well as motion detection and AI-based training guidance algorithms, the HUAWEI TruSportTM scientific training system provides a reference for assessing the running ability, training load, and physical condition of amateur runners. The fundamental principle behind the performance prediction of the system is that after completing the initial run, the watch can display the predicted results for distances of 5 km, 10 km, half-marathons, and full marathons based on the individual's heart rate, speed measurements, and the relationship between the two. Every 24 h, regardless of whether a run has taken place on that day, the watch updates the performance predictions by connecting to the APP. If no exercise is performed on a particular day, the changes in performance predictions are minimal and can be ignored. However, if a run is completed on a given day, the performance predictions are refreshed based on factors such as the heart rate, time, and running speed. According to the official instructions for the HUAWEI WATCH GT Runner smartwatch, runners should wear the smartwatch for 42 days for a more accurate prediction of running performance.The HUAWEI TruSportTM scientific training system updates the performance predictions based on all the running data within each 42-day cycle. This process incorporates rolling iterations; therefore, the performance prediction on the 43rd day is updated using complete data from days 1–42, the 44th day prediction is based on days 2–43, and so on. Consequently, after 43 days of wearing the smartwatch, the running performance predictions consistently utilize a rolling update based on the previous 42 days of data, rather than relying solely on the most recent run. This mitigates the impact of short-term fluctuations in running performance on the prediction. Therefore, to assess runners' ability more accurately, the participants wore the watch for at least 42 days.

### 2.4 Data analysis

Statistical analysis was performed using SAS JMP Pro 17.0.0. Descriptive statistics were used to analyze the participants' basic information. Several methods were employed to assess the accuracy of the watch's predicted scores. A simple correlation coefficient (*r*) was used to describe the correlation between the measured and predicted scores. The coefficient of determination (*r^2^*) was used to assess the goodness of fit between the measured and predicted scores. The absolute error rate was calculated using the formula (measured value—predicted value)/measured value × 100%. An error rate less than 5% was considered acceptable. The intraclass correlation coefficient (ICC) was used to evaluate the consistency between the predicted and measured values. The corresponding standards for ICC, indicating excellent, good, fair, and poor agreement, were ≥0.9, 0.75–0.9, 0.6–0.75, and ≤0.75, respectively. To evaluate the level of agreement between the predicted and measured values, the limits of agreement (LoA) interval was calculated by adding and subtracting 1.96 times the standard deviation from the difference between the measured and predicted values. The Bland–Altman scatter plot was employed to visually describe the agreement, with a consistency rate of over 95% within the LoA interval indicating good agreement (10, 18).

## 3 Results

The measured and predicted values for the 5 km, 10 km, and half-marathon (21.1 km) distances showed a high degree of correlation (*r* ≥ 0.95, *p* < 0.001) (see Table 2) and *r^2^* ≥ 0.9, indicating a strong goodness of fit between the measured and predicted values. The error rate between the measured and predicted values was less than 3%, with an ICC ≥ 0.9, indicating a high level of consistency between the predicted and measured values. For the 5 km distance, the bias ± 95% LoA was −20.4 ± 44.2 s. For the 10 km distance, the bias ± 95% LoA was 4.1 ± 299.1 s. Finally, for the half-marathon distance, the bias ± 95% LoA was 143.8 ± 400.4 s.

**Table 2:** Correlation between measured performance and predicted performance.

TD	SS	MP(sec)	PP(sec)	ER(%)	*R*	*r* ^2^	SEE (sec)	ICC	L LOA	U LOA
5 km	13	1,382.3 ± 141.3	1,402.9 ± 145.9	−1.47	0.99	0.98	32.9	0.98	−64.6	23.8
10 km	237	2,999.9 ± 528.2	2,935.6 ± 485.6	0.10	0.95	0.90	153.0	0.96	−295.0	303.2
21.1 km	38	6,015.9 ± 667.0	5,872.1 ± 632.0	2.30	0.95	0.91	254.4	0.93	−256.6	544.1
Total	288	3,292.4 ± 1,179.5	3,253.9 ± 1,180.2	0.32	0.99	0.98	165.6	0.99	−299.8	342.7

Values are mean ± SD. TD, Test Distance; SS, Sample size; MP, Measured performance; PP, Predicted Performance; ER, Error Rate; ICC, intraclass correlation coefficient; LOA, The Limits of Agreement.

**Figure 1:**
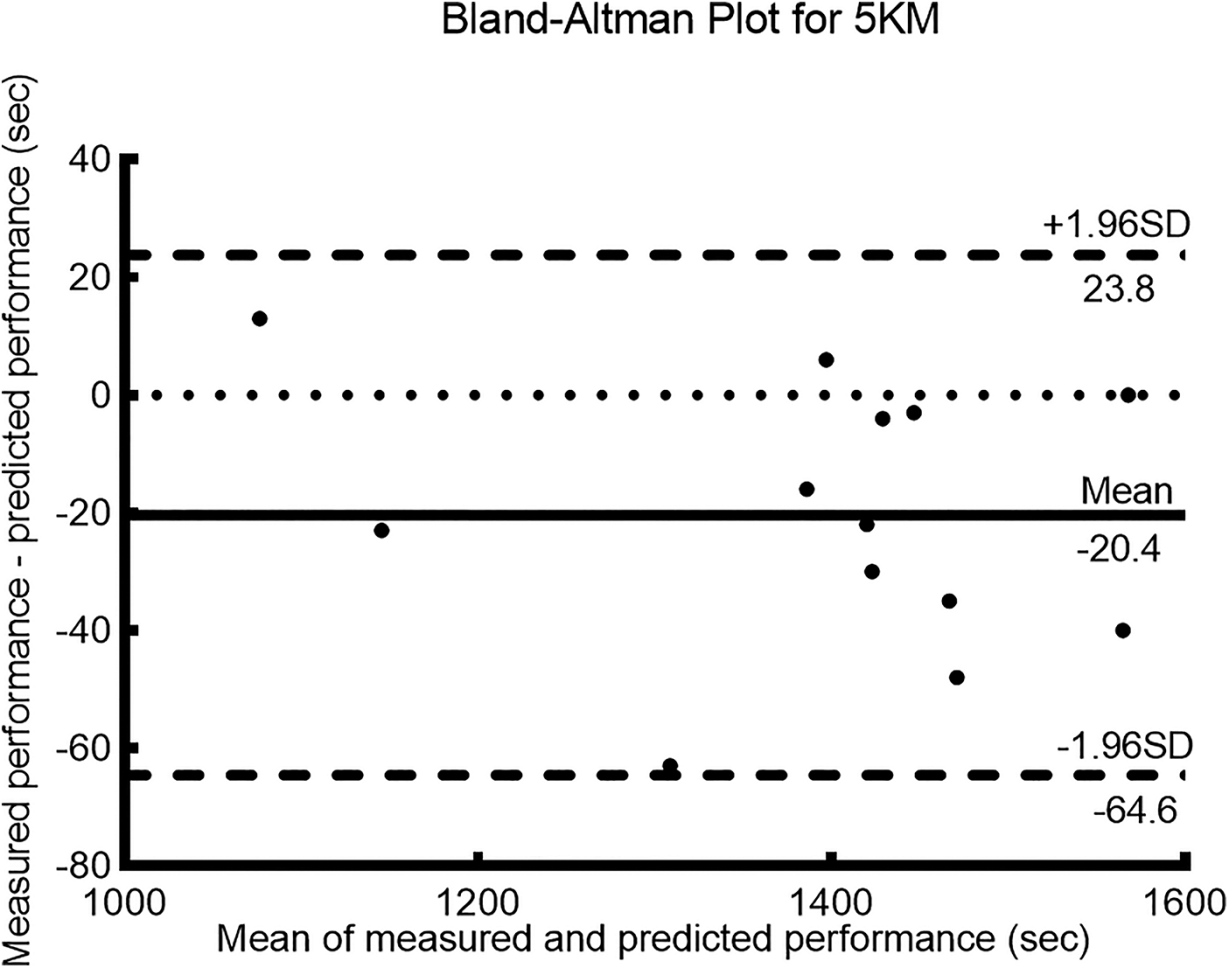
Bland-Altman plots of measured and predicted performance for the 5 km run.

**Figure 2:**
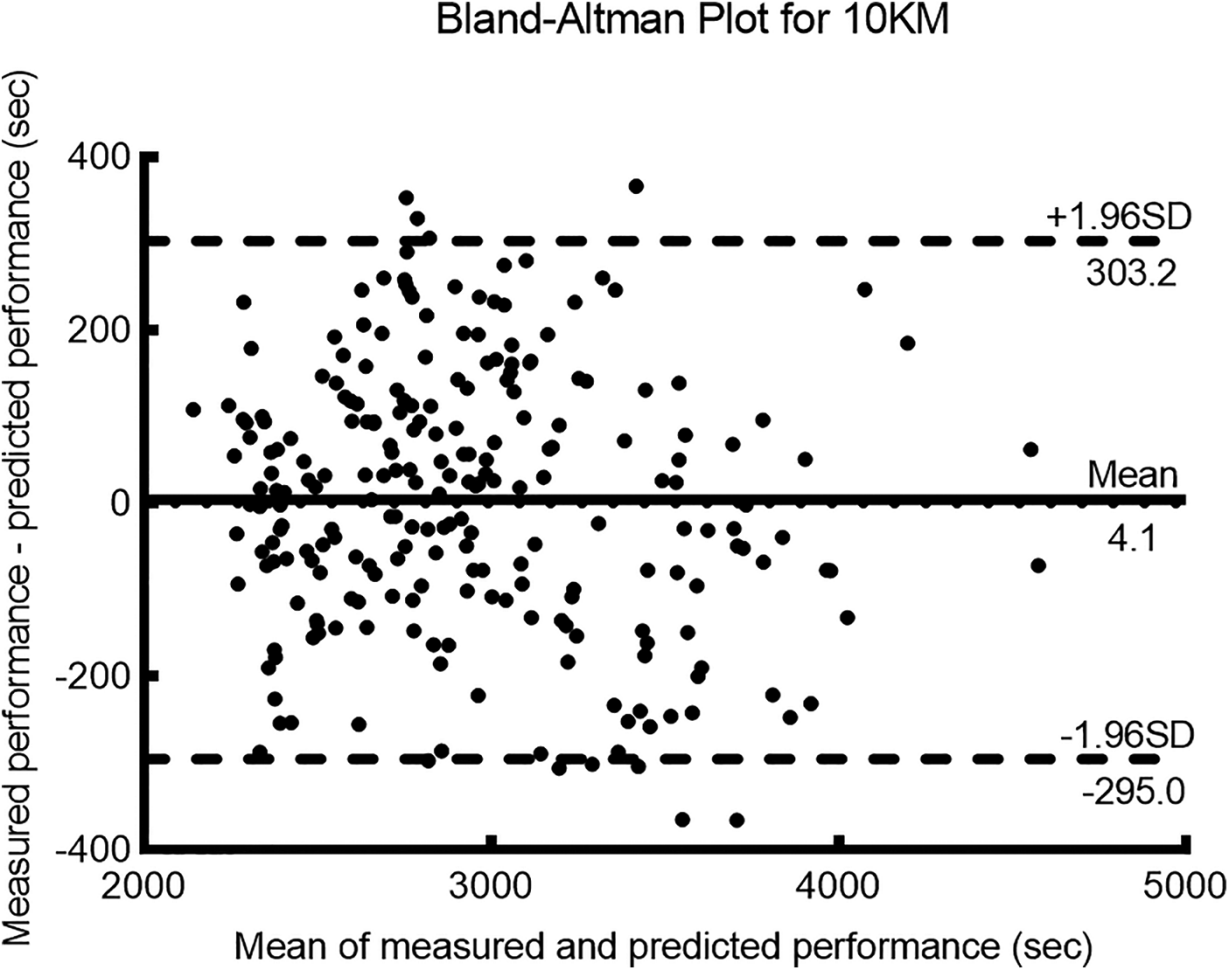
Bland-Altman plots of measured and predicted performance for the 10 km run.

**Figure 3:**
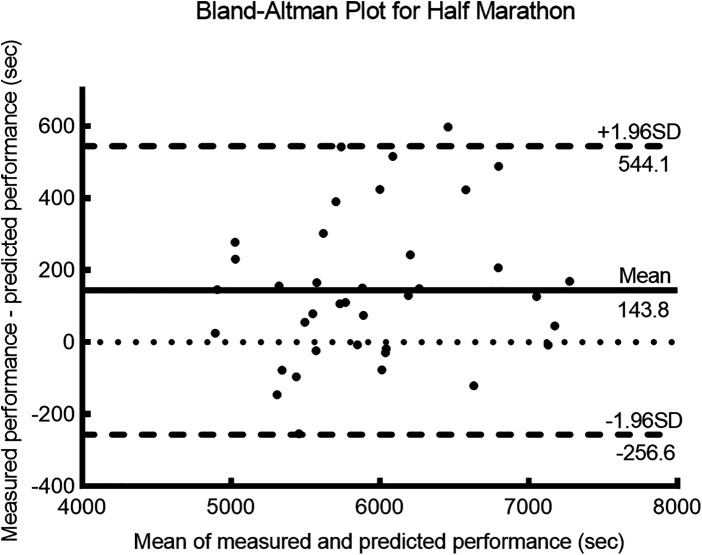
Bland-Altman plots of measured and predicted performance for the half-marathon (21.1 km) run.

Figures 1–3 present the Bland–Altman scatter plots for the 5 km, 10 km, and half-marathon distances, respectively. In these plots, 100% of the scatter points for the 5 km test, 95.8% of the scatter points for the 10 km test, and 97.4% of the scatter points for the half-marathon test fall within the bias ± 95% LoA interval.

## 4 Discussion

The purpose of this study was to validate the accuracy of a smartwatch (HUAWEI WATCH GT Runner) for predicting running performance. The participants' percentage of maximum heart rate and RPE results (Table 1) during the 5, 10, and 21.1 km tests indicated that they performed the tests with maximum effort (17, 19). Based on the results of this study, the hypothesis was confirmed. The findings of this study revealed a high correlation between the measured and predicted performances for the 5 km, 10 km, and half-marathon distances (*r* = 0.99, *r* = 0.95, and *r* = 0.95, respectively). The ICCs for all distances exceeded 0.9, and the Bland–Altman analysis showed that over 95% of the differences fell within the LoAs, indicating good consistency. The error rates for all three distances were below 3%, with the lowest error rate observed for the 10 km distance at 0.10%. The coefficient of determination (*r^2^*) for the 10 km distance was 0.90, and the bias ± 95% LoA was 4.1 ± 299.1 s. The error rate for the half-marathon predicted performance was 2.3%, with an *r^2^* of 0.91, and the bias ± 95% LoA was 143.8 ± 400.4 s. Therefore, the errors in the performance predicted by the smartwatch were deemed acceptable, with a higher accuracy observed in the prediction of the 10 km performance than the half-marathon.

In previous studies, various mathematical models have been employed to predict running performance. These models include linear, logarithmic, hyperbolic, and multiple regression models (20–22). Correlations between aerobic parameters (VO2max, vVO2max, and LT), training variables (training volume and pace), body composition indicators (body mass index and body fat percentage), and running performance were analyzed. Regression models were used to establish the prediction equations. Among these models, the prediction models for marathon performance based on body morphology and training variables demonstrated moderate utility with *r^2^* values ranging from 0.41 to 0.68 (12, 23). Beat Knechtle predicted half-marathon times for male and female runners using the following equations: male race time (in min) = 142.7 + 1.158 × body fat percentage (%)–5.223 × running speed during training (km/h) (*r^2^* = 0.41); and female race time (in min) = 168.7 + 1.077 × body fat percentage (%)–7.556 × running speed during training (km/h) (*r^2^* = 0.68) (11, 24, 25). However, higher accuracy in predicting running performance has been achieved by combining training variables with aerobic parameters (*r^2^* = 0.87–0.90) (24). Gómez used parameters such as the respiratory compensation threshold (RCT) speed, vVO2max, training experience, weekly training volume, stride length, and stride frequency to establish four prediction models. The model based on RCT speed, vVO2max, and training experience demonstrated the highest relative accuracy (*r^2^* = 0.90) for half-marathon prediction. The predicted time (in min) for this model was calculated as follows: predicted time (min) = 169.54–2.51 peak speed (km/h)–2.25 RCT speed (km/h)–0.37 running experience (years) (24).

In recent years, with the rapid development of technology, the use of big data and intelligent algorithms has made it possible to improve the accuracy of performance prediction. Intelligent performance prediction algorithms based on AI include the Artificial Neural Network (ANN), k-Nearest Neighbor (KNN), local matrix completion, and others (26–28). Prediction algorithms based on AI differ from mathematical models in that they involve premodeling, continuous learning, and the refinement of algorithms based on the collected human and training data, improving their accuracy as the sample size increases. In a study that utilized variables such as the underlying 10 km performance, body mass index, age, and sex, ANN and KNN models were used to predict marathon performance. The results showed that the correlation coefficients of both models reached 0.9, and the prediction accuracies were above 94% (with KNN outperforming ANN with an average absolute error of 2.4% compared with 5.6% for ANN), indicating high accuracy (18). The results demonstrated high precision in predicting running performance via mathematical models or AI-based intelligent algorithms. This indicates that predicting running performance has matured in terms of logical reasoning and computational models in experimental research, thereby providing a theoretical basis for designing and developing wearable smart devices to predict running performance.

Our research results demonstrated that the accuracy of performance prediction by the smartwatch was above 97%, indicating satisfactory precision in performance prediction by the smartwatch. According to Huawei's official website, the smartwatch uses the HUAWEI TruSportTM system to predict running performance, but the algorithm for predicting running performance and specific related variable indicators is not publicly indicated in the product description, which may involve trade secrets (29). According to the introduction of the watch function, the smartwatch uses distance measurement through GPS technology and heart rate measurement through PPG technology during running, and these two technologies are currently recognized as mature technologies for smartwatches to measure distance and heart rate, so the input parameters are consistent and rigorous, and at the same time, they provide reliable data for the accurate prediction of running performance. Heart rate, running pace, and running volume are commonly used indicators for predicting running performance. Heart rate and running pace are utilized to assess training intensity, while running volume evaluates training load. These indicators are then incorporated into prediction models to generate performance outcomes. Hagan developed marathon performance prediction models based on running pace and running volume, which demonstrated high accuracy: Race Time = 449.88−7.61 (Mean km/day)−0.63 (Training pace, m/min) (*R*^2^ = 0.68) and Race Time = 214.24 + 393.07 (BMI)−0.68 (Training pace, m/min) (*R*^2^ = 0.76) (30).

Research has also confirmed that predicting running performance using data from races that are closer in distance yields higher accuracy (25). The HUAWEI WATCH GT Runner refreshes performance predictions based on each running performance and updates them based on past running performance, aligning with the inherent relationship between training level and performance, thus ensuring the reliability of the indicators.

In this study, the smartwatch exhibited high accuracy in predicting performance at 5 km, 10 km, and half-marathon distances, with an accuracy rate exceeding 97%. Therefore, for amateur runners, the long-term use of the smartwatch can provide accurate predictions of race performance and assist runners in training more effectively and formulating race strategies based on these predictions.

### 4.1 Limitations of the study

First, the participants in this study were amateur runners rather than high-level athletes. Further validation is required to determine whether the smartwatch can accurately predict the performance of elite athletes. Second, we observed a discrepancy in sample sizes across the different testing distances, with a larger concentration of samples in the 10 km and half-marathon tests, whereas the sample size for the 5 km test was relatively small.

### 4.2 Practical application

Aerobic metabolism indicators such as VO2max, vVO2max, and lactic threshold can be used to predict long-distance running performance. However, obtaining these indicators requires expensive laboratory equipment and stringent experimental conditions, making it difficult for amateur runners to access the testing opportunities. The HUAWEI WATCH GT Runner is a running smartwatch designed for the public. By continuously recording metrics such as heart rate and running speed, time, and distance, it provides accurate and dynamic information for predicting running performance. The smartwatch offers the advantages of simpler operation, shorter time, and lower economic costs compared with traditional laboratory testing based on aerobic metabolism indicators, making it highly promising for practical applications.

## Data Availability

The datasets presented in this study can be found in online repositories. The names of the repository/repositories and accession number(s) can be found in the article/Supplementary Material.
